# Successful Coil Embolization of a Giant Celiac Artery Aneurysm in a Patient With Liver Cirrhosis: A Case Report

**DOI:** 10.7759/cureus.99871

**Published:** 2025-12-22

**Authors:** Kunitaka Kumagai, Shohei Takasugi, Yoshiharu Nishibori, Motonobu Nishimura

**Affiliations:** 1 Vascular Surgery, Kawasaki Hospital, Kobe, JPN; 2 Radiology, Tottori University Hospital, Yonago, JPN; 3 Cardiology, Kawasaki Hospital, Kobe, JPN

**Keywords:** 3dct, celiac artery aneurysm, cirrhosis liver, coil embolization, replaced right hepatic artery

## Abstract

In patients with uncompensated liver cirrhosis, celiac artery embolization poses the risk of hepatic ischemia, which may lead to liver failure. We report a case of coil embolization of a giant celiac artery aneurysm in a 63-year-old man with liver cirrhosis. Preoperative contrast-enhanced and three-dimensional (3D) computed tomography (CT) angiography demonstrated a replaced right hepatic artery arising from the superior mesenteric artery and well-developed collateral circulation to the proper hepatic artery via the pancreaticoduodenal arcade. Based on these findings, coil embolization of the celiac artery was performed under local anesthesia. Intraoperative angiography with temporary balloon occlusion of the celiac artery confirmed adequate collateral hepatic circulation via the superior mesenteric artery before the embolization. Final angiography demonstrated complete elimination of contrast enhancement within the aneurysm. The operative time was 106 minutes, and blood loss was minimal. The postoperative course was uneventful, with no worsening of liver function. Follow-up CT performed one year after the procedure demonstrated aneurysm shrinkage, with the diameter decreasing from 60 × 67 mm to 46 × 50 mm, without progression of liver dysfunction. This case highlights the importance of detailed preoperative vascular evaluation using 3D CT to safely perform celiac artery embolization in patients with liver cirrhosis.

## Introduction

The most common visceral artery aneurysm is the splenic artery aneurysm, accounting for 60% of cases, while celiac artery aneurysms are relatively rare, accounting for 4% of cases [1]. Therefore, comprehensive reports on celiac artery aneurysms are limited [2]. Celiac artery embolization is a well-established endovascular technique and some reports have documented its efficacy in the treatment of celiac artery aneurysms [3]. However, treatment of celiac artery aneurysms in patients with uncompensated liver cirrhosis is extremely rare, and to the best of our knowledge, reports of celiac artery coiling for such cases have been identified only in an abstract form [4]. In patients with celiac artery aneurysms complicated by cirrhosis, embolization of the celiac artery may cause serious complications [5]. Here, we present a case of successful coil embolization of a giant celiac artery aneurysm in a patient with liver cirrhosis, in which preoperative three-dimensional (3D) computed tomography angiography (CTA) was done for evaluation of the hepatic arterial anatomy and collateral circulation.

## Case presentation

A 63-year-old man with untreated hypertension presented with abdominal distension and loss of appetite. The patient drank three liters of whiskey and soda every day, and his alcohol intake was estimated to exceed 170 g/day. He was alert, and there were no signs of encephalopathy. Physical examination revealed conjunctival icterus and bilateral lower-limb edema. Computed tomography (CT) revealed a celiac aneurysm with a diameter of 69 mm, liver surface irregularities, and moderate ascites. His laboratory results are summarized in Table 1. 

**Table 1:** Preoperative laboratory parameters and their reference ranges AST: aspartate transaminase; ALT: alanine transaminase; γ-GT: gamma-glutamyl transferase; BUN: blood urea nitrogen; CRP: C-reactive protein; BNP: B-type natriuretic peptide; WBC: white blood cell count; PT-INR: prothrombin time-international normalized ratio.

Laboratory test	Result	Reference range	Unit
Sodium	135	137-146	mEq/L
Potassium	3.7	3.6-5.4	mEq/L
Total bilirubin	2.8	0.2-1.2	g/dL
Albumin	3.2	3.9-4.9	g/dL
AST	180	2-40	U/L
ALT	42	0-35	U/L
γ-GT	939	0-55	U/L
Amylase	23.4	40-126	mg/dL
BUN	8.9	7-20	mg/dL
Creatinine	0.83	0.61-1.04	mg/dL
CRP	0.6	0-0.3	mg/dL
BNP	57.3	0-100	pg/mL
Hepatitis B surface antigen	<0.001	<0.0049	IU/mL
Hepatitis C virus antibodies	0.1	0-0.9	C.O.I
WBC	6300	3300-8600	/µL
Hemoglobin	11.2	13.5-17.0	g/dL
Platelet count	9	15-35	× 10^4^/µL
PT-INR	1.04		
D-dimer	5.9	0-1.0	µg/mL

The presence of moderate ascites and the laboratory results corresponded to a Child-Pugh score of nine (Class B), indicating decompensated cirrhosis [6]. The Model for End-Stage Liver Disease (MELD) and Model for End-Stage Liver Disease incorporating serum sodium (MELD-Na) scores were eight and 12, respectively [7,8].

The liver dysfunction was attributed to heavy alcohol consumption. Because of its large size (>6 cm), the celiac aneurysm was considered to have a high risk of rupture, and treatment was mandatory. Although catheter embolization is widely used to treat celiac artery aneurysms, the patient also had liver cirrhosis. Common hepatic artery embolization raised concerns regarding the progression of liver dysfunction due to decreased hepatic blood flow.

**Figure 1:**
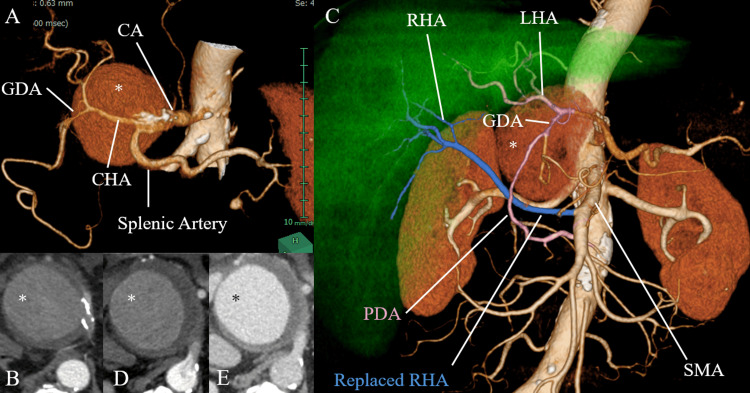
Preoperative CT findings Asterisks indicate celiac artery aneurysm. (A, B) Celiac artery aneurysm arising from a calcified celiac artery; (C) Three-dimensional reconstruction image of hepatic circulation; (D) Retrograde blood flow in the celiac artery during the arterial phase; (E) Contrast enhancement of the celiac artery in the venous phase. CA: celiac artery; CHA: common hepatic artery; GDA: gastroduodenal artery; RHA: right hepatic artery; LHA: left hepatic artery; SMA: superior mesenteric artery; PDA: pancreaticoduodenal arcade.

To determine the optimal treatment strategy, the visceral arteries were assessed in detail using CT angiography (CTA) images. CTA revealed a saccular aneurysm of the celiac artery (Figures 1A, 1B).

The diameter of the common hepatic artery and celiac artery was 3.5 mm and 9 mm, respectively. A reconstructed 3D image revealed that the right hepatic artery arose from the superior mesenteric artery, a so-called replaced right hepatic artery (RHA), and anastomosis from the superior mesenteric artery (SMA) to the proper hepatic artery via the pancreaticoduodenal arcade (PDA) (Figure 1C). Moreover, an arterial phase CTA showed good contrast enhancement, excluding the origin of the celiac artery (Figures 1A, 1C). The origin of the celiac artery was not contrasted during the arterial phase (Figure 1D) but was well contrasted during the venous phase (Figure 1E), and no significant stenosis was observed in it. Contrast defects were observed in the aorta at the level of the celiac artery (Figure 1D), suggesting retrograde filling of the celiac artery due to the giant aneurysm. The CT attenuation value of the aneurysmal lumen increased from 93 Hounsfield units (HU) in the arterial phase to 156 HU in the portal venous phase. These findings suggested decreased antegrade blood flow in the celiac artery, possibly caused by a giant aneurysm. Therefore, coil embolization was performed.

**Figure 2:**
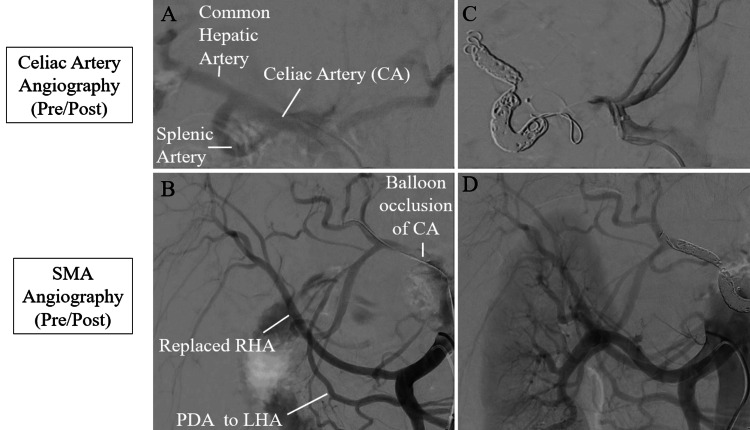
Operative angiography (A) Preoperative celiac artery (CA) angiography; (B) Preoperative superior mesenteric artery (SMA) angiography with balloon occlusion of the CA; (C) Postoperative CA angiography; (D) Postoperative SMA angiography. CA: Celiac artery; SMA: superior mesenteric artery; RHA: right hepatic artery; LHA: left hepatic artery; PDA: pancreaticoduodenal arcade.

Under local anesthesia, the bilateral femoral arteries were punctured, and 5-Fr sheaths (Terumo Corporation, Tokyo, Japan) were inserted. The artery scheduled for embolization was confirmed using angiography of the celiac artery distal to the aneurysm (Figure 2A).

As predicted based on the preoperative diagnosis, SMA angiography with balloon occlusion of the celiac artery root demonstrated collateral circulation to the right and left hepatic arteries via the SMA (Figure 2B).

First, the splenic artery was embolized using POD coils (Medicos Hirata, Osaka, Japan): 8 mm × 60 cm and 6 mm × 50 cm, and EMBOLD fibered coils (Boston Scientific, Marlborough, MA, USA): 4 × 15 cm, 5 × 15 cm, and 5 × 15 cm. Second, the common hepatic artery was embolized using a POD coil 4 mm × 30 cm and a POD packing coil (45 cm). Finally, the root of the celiac artery was embolized using an Amplatzer Vascular Plug II (AVP II; 12 mm, Abbott, Abbott Park, IL, USA) (Figure 2C). Coil embolization was performed using an RC1 catheter (4 Fr, Medikit, Tokyo, Japan), a Leonis Mova steerable microcatheter (2.9 Fr, Sumitomo Bakelite, Tokyo, Japan), a Carnelian microcatheter (1.9 Fr, Tokai Medical Products, Aichi, Japan), and a Meiter microwire (0.016 inch and 0.014 inch, Asahi Intecc, Aichi, Japan).

No aneurysm was detected on contrast imaging of the celiac artery root or SMA (Figures 2C, 2D). Finally, contrast imaging of the SMA was used to confirm collateral circulation to the right and left hepatic arteries via the SMA (Figure 2D), and endovascular treatment was completed. The operative time was 106 min, and there was minimal blood loss.

Laboratory test results on postoperative days zero and one showed no progression of liver dysfunction (Table 2), and the patient was discharged on postoperative day one without complications. 

**Table 2:** Postoperative course of the laboratory data AST: aspartate transaminase; ALT: alanine transaminase; γ-GT: gamma-glutamyl transferase; BUN: blood urea nitrogen; CRP: C-reactive protein; BNP: B-type natriuretic peptide; WBC: white blood cell count; PT-INR: prothrombin time-international normalized ratio.

Laboratory test	postoperative day 0	postoperative day 1	1 year after surgery	Reference range	Unit
Sodium	131	132	140	137-146	mEq/L
Potassium	3.1	3	3.5	3.6-5.4	mEq/L
Total bilirubin	1.6	1.8	1.5	0.2-1.2	g/dL
Albumin	2.9	2.9	3.1	3.9-4.9	g/dL
AST	145	130	157	2-40	U/L
ALT	34	35	28	0-35	U/L
γ-GT	592	595	425	0-55	U/L
Amylase	135	N/A	N/A	40-126	mg/dL
BUN	9.1	8.2	10.6	7-20	mg/dL
Creatinine	0.62	0.67	1.34	0.61-1.04	mg/dL
C-reactive protein	0.6	0.6	0.2	0-0.3	mg/dL
WBC	3600	3800	5400	3300-8600	/µL
Hemoglobin	9.8	10.7	9.5	13.5-17.0	g/dL
Platelet count	9	9	14	15-35	× 10^4^/µL
PT-INR	1.36	1.07	1.11		
D-dimer	5.5	N/A	6.4	0-1.0	µg/mL

**Figure 3:**
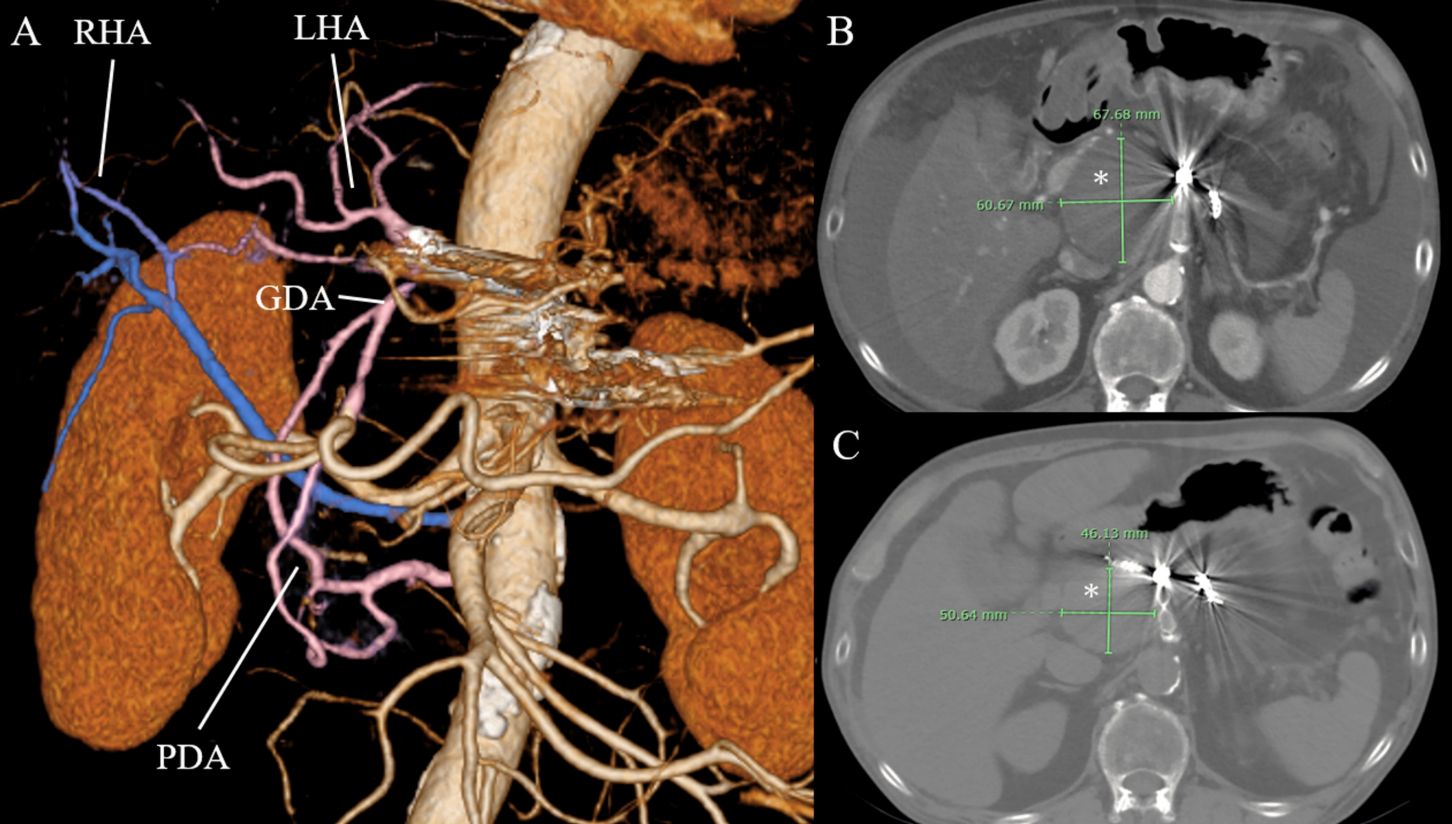
Postoperative CT findings. Asterisks indicate celiac artery aneurysm. (A) CT image 1 month after surgery; (B) Diameter of the celiac artery aneurysm 1 month after surgery; (C) Diameter of celiac artery aneurysm 1 year after surgery. RHA: right hepatic artery; LHA: left hepatic artery; GDA: gastroduodenal artery; PDA: pancreaticoduodenal arcade.

CTA performed one month after surgery revealed the left and right hepatic arteries and disappearance of contrast enhancement within the aneurysm (Figure 3A).

Comparison of the CT scans obtained one month and one year after surgery revealed a reduction in aneurysm diameter from 60 × 67 mm to 46 × 50 mm (Figures 3B, 3C).

Laboratory tests one month after surgery remained stable with no progression of liver dysfunction (Table 2). The patient is currently being followed-up with CT scans every six months.

## Discussion

According to the 2020 Society for Vascular Surgery (SVS) guidelines, endovascular intervention is recommended for elective celiac artery aneurysm treatment if it is anatomically feasible [3]. However, for elective treatment, any of the following methods - open surgical, endovascular, or laparoscopic - may be appropriately selected based on the patient's anatomy and underlying clinical condition. Embolization of the celiac artery is generally performed to treat aneurysms of the celiac artery. It is also performed during thoracic endovascular repair, in which a thoracic aortic stent graft must be placed over the orifice of the celiac artery. Embolization of the celiac artery is well tolerated in most cases but is a problem in patients with underlying hepatic disease [9].

In this case, the patient had cirrhosis; therefore, various treatment approaches were considered. We first considered using a covered stent to preserve the antegrade blood flow in the celiac artery [10,11]. However, it was deemed unsuitable owing to a significant mismatch in diameter between the celiac artery and common hepatic artery. Second, we considered open surgery to preserve the celiac antegrade blood flow. Surgical options include graft interposition, or bypass surgery using an autologous saphenous vein or prosthetic graft. However, given the complexity of surgery due to the large size of the aneurysm and the risk of complications from moderate ascites accumulation, we determined that a less invasive treatment approach should be selected.

CTA is extremely helpful in performing celiac artery embolization to evaluate the collateral circulation to the liver. We carefully reviewed preoperative contrast-enhanced CT images and confirmed the presence of adequate collateral circulation. In this case, the first collateral circulation was the presence of the replaced RHA [12]. Even when the common hepatic artery was occluded, RHA blood flow was preserved. The second collateral circulation was the anastomosis from the SMA to the left hepatic artery via the PDA. Therefore, even if the common hepatic artery is occluded, left hepatic artery blood flow is preserved by collateral circulation from the SMA. We performed a final confirmation during the catheter intervention. Prior to embolization, we confirmed contrast flow into both hepatic arteries via SMA angiography, while the celiac artery was occluded with a balloon [13]. Based on these results, we concluded that the embolization could be safely performed.

Considering hepatic blood flow, covered stent placement is preferable to embolization; however, this approach is plagued by anatomical limitations and the requirement for postoperative oral antiplatelet therapy. In cases where sufficient collateral circulation is expected, simple embolization may be an acceptable treatment of choice rather than revascularization.

## Conclusions

A patient with liver cirrhosis underwent embolization from the common hepatic artery to the celiac artery without any deterioration in liver function. In this case, a replaced RHA arising from the SMA was considered an important collateral circulation route after embolization.

In patients with liver dysfunction, treatment without obstructing hepatic blood flow is preferable. However, if a preoperative 3D-CT scan identifies vessels that can provide sufficient collateral circulation, such as a replaced RHA, embolization may be a viable option even in patients with liver dysfunction.
